# Registered general nurses' health assessment practices in a tertiary hospital: A focused ethnography study

**DOI:** 10.1002/nop2.2237

**Published:** 2024-07-03

**Authors:** Mohammed Awal Salifu, David Abdulai Salifu, Janet Gross

**Affiliations:** ^1^ Nursing and Midwifery Training College Damongo Ghana; ^2^ School of Nursing and Midwifery University for Development Studies Tamale Ghana; ^3^ Mother Patern College of Health Sciences Stella Maris Polytechnic University Monrovia Liberia

**Keywords:** focused ethnography, nurses, nursing health assessment

## Abstract

**Aim:**

To explore the assumptions and values that influence nursing health assessment practices among registered general nurses in general medical and surgical wards.

**Design:**

The study was designed as a focused ethnography.

**Methods:**

A semi‐structured interview guide was used to explore prevailing nursing health assessment practices of 13 registered general nurses in an attempt to explore the assumptions and values influencing health assessment practices in the study setting. Data were analysed inductively using an interpretive qualitative content analysis method.

**Results:**

Nursing health assessment practices, and underlying assumptions and values were underpinned by a central theme of a culture of low expectation relating to nursing health assessment. The culture of low expectation was highlighted in five themes: (1) Unsystematic Assessment of Health Status, (2) Purpose of Nursing Health Assessment, (3) The Role of Nursing Educational and Regulatory Institutions, (4) Ward Ethos and (5) The Role of Organizational and Ward Leadership.

**Implication:**

The adoption of a holistic nursing health assessment framework with a clearly defined purpose of aiding nursing diagnoses can guide patient‐centred care delivery and facilitate early recognition of physiological deterioration.

**Patient or Public Contribution:**

Thirteen registered general nurses were interviewed, and the initial findings returned to them for validation.

**Conclusion:**

The potential contribution of nursing health assessment to nursing practice and patient outcomes may not be fully realized if nursing health assessment is not situated within a holistic health assessment model with a clearly defined purpose for nursing practice.

## 1 INTRODUCTION

Nursing health assessment (NHA) is conceptualized as an essential component of clinical nursing practice. It is a broad term describing the use of the skills of history taking and physical assessment to collect both subjective and objective data on health behaviours and functioning of an individual, family or community (Carpenito, 2021). When incorporated into a holistic model of care, NHA data provide the basis for diagnosing and treating complex alterations in functional health responses, and the delivery of patient‐centred nursing care (Herdman et al., 2021). There is also evidence suggesting that NHA data may also be used collaboratively to contribute to medical diagnostic decision‐making, and to identify and prevent deterioration among hospital in‐patients (Zambas et al., 2016). A thorough NHA is therefore an unquestionable prerequisite to the successful fulfilment of clinical nursing roles. Notwithstanding the integral function of NHA to the fulfilment of clinical nursing roles, nurses' utilization of health assessment skills has consistently been underwhelming.

## 2 BACKGROUND

The skill of history taking is the single most important component of patient health assessment and provides the most evidence for making an informed clinical judgement. Within the nursing profession, as important as it may be, a universally acknowledged and widely adopted history‐taking framework that fits perfectly into the philosophy of nursing is non‐existent. A review of the literature on nursing history‐taking frameworks shows a shift in focus from a medically inclined approach (Fawcett & Rhynas, 2012; Lloyd & Craig, 2007) to a more biopsychosocial and humanistic approach to nursing health history taking (Wilson & Giddens, 2020). Although recommended as the guiding framework for NHA, the Functional Health Patterns (FHPs) proposed by Gordon (2008) have not been fully utilized. These patterns provide a logical, comprehensive coverage of the different dimensions of health and human functioning. A number of health screening tools based on the FHPs have been validated for use by nurses in clinical settings (Gengo E Silva Butcher & Jones, 2021) but have not been widely adopted for routine clinical use. The thinking that NHA is unstructured, accumulative and invisible (Fawcett & Rhynas, 2012; Zambas, 2010) may explain the lack of a universal and widely accepted nursing history‐taking framework.

Research on nurses' health assessment practices has focused largely on quantifying the frequency of use of various physical assessment skills and the factors that promote or inhibit the use of these skills. The frequency of use of physical assessment skills in everyday practice among nurses has been found to be low and tends to include mainly the technique of inspection (Tan et al., 2021). This trend of physical assessment skills use, interestingly, has no significant correlation with the age, gender, level of education or years of clinical experience of Registered Nurses (RNs) (Fennessey, 2016; Giddens, 2006). Although the barriers to nurses' use of physical assessment skills is complex and diverse, lack of knowledge and confidence in performing physical assessment skills appears as a common theme among nursing students and RNs (Maniago et al., 2021; Tan et al., 2021). Other factors identified as barriers to nursing students' use of physical assessment skills include lack of nursing role models, limited practice opportunities and the theory‐practice gap (Maniago et al., 2021). Among RNs, Tan et al. (2021) emphasized role ambiguity and lack of influence on patient care, reliance on others and technology, lack of time and interruptions, ward culture and variation across care specialties. Available research on nurses' health assessment practices and associated barriers has largely utilized quantitative methods, and does not provide sufficient explanation for the low frequency of use of physical assessment skills, and the assumptions and values underpinning the practice and barriers identified. The low utilization of physical assessment skills coupled with the lack of a significant correlation between physical assessment skills use and personal characteristics of nurses may suggest the existence of a professional culture or subculture influencing nurses' health assessment practices.

In order to enhance our understanding of the assumptions and values tacitly influencing nursing action and behaviour, and to design impactful behaviour change interventions, there is the need to examine nursing practice as a culture or subculture (Hartrick Doane & Varcoe, 2005). No study, to the best of our knowledge, has explored nurses' health assessment practices as a cultural or subcultural phenomenon using qualitative methods.

## 3 THE STUDY

### 3.1 Aim and objective

To explore the assumptions and values that underpin NHA practices among registered general nurses in medical or surgical wards, and how these assumptions and values influence the nurses' health assessment practices. This study also describes the NHA practices in the study setting.

## 4 METHODS

### 4.1 Design

A focused ethnographic design is fit for a research endeavour interested in exploring and describing subcultures, and the constitution of a shared behaviour among a subgroup of society (Cruz & Higginbottom, 2013; Higginbottom et al., 2013). This methodology applies ethnographic methods to the study of a focused and distinct phenomenon or shared behaviour (i.e. NHA practices) within a culture or subculture (i.e. nursing, organizational or ward culture) of a people (i.e. registered general nurses).

### 4.2 Theoretical framework

We conceptualized practices of nurses' health assessment as a cultural artefact and a product of the subculture of general wards. Culture plays an important role in guiding the thinking, decision‐making and action of a group through the influence of shared assumptions, values and beliefs (Schein, 2010). We believe that every ward has a unique culture but there may also be similarities in culture across wards within the same healthcare organization. We adopted the Cultural Dynamics Model (Hatch, 1993) as the theoretical framework for this study. This model, rooted in symbolic interpretation, recognizes four domains of culture including assumptions, values, artefacts and symbols (Hatch, 1993). Cultural assumptions refer to beliefs about reality and human nature which often, unconsciously, influence values. Cultural values describe the basic social principles and standards, whiles cultural artefacts are the visible and tangible results of behaviour or activity rooted in values and assumptions.

Cultural domains are enacted through four processes including manifestations, realizations, symbolization and interpretation (Hatch, 1993). Cultural manifestation describes the processes underlying the translation of assumptions into values and vice versa. Cultural realization focuses on processes through which values and expectations find expression in an activity resulting in a social or material reality with a tangible outcome. Values and expectations may also be realigned if the artefact experienced by members of the culture is in dissonance with previously held values and expectations (Hatch, 1993). The scope of this study is limited to the cultural domains of assumptions and values, and the cultural processes of manifestation and realization.

### 4.3 Study setting

The study was conducted at a teaching hospital serving as a major referral centre for the northern sector of the country. The hospital provides advanced clinical healthcare services, partakes in the training of healthcare professionals and conducts research for healthcare improvement. The hospital has a total bed capacity of about 800 and a staff strength of about 1259 nurses and 342 medical doctors. The average length of stay in hospital for the year 2019 was 6.7 days.

### 4.4 Inclusion and exclusion criteria

The target population for this study included all Registered General Nurses (RGNs), irrespective of years of clinical experience, working in a general medical or surgical ward. RGNs and specialty nurses working in specialty units such as accident and emergency, paediatric, labour and maternity and intensive care units were considered as ineligible as the focus of the study was on NHA practices among RGNs in general medical or surgical wards. Nurse managers of the wards included in the study together with the director of nursing service of the hospital were also interviewed.

### 4.5 Sampling and recruitment

A purposive sampling approach, complemented by solicitations, was used to recruit nurse managers and RGNs working in a general medical or surgical ward for semi‐structured in‐depth interviews. Ward managers were asked to identify RGNs who met the inclusion criteria and were capable of providing rich data towards the aim of the study. Solicitation adopts the extension of a ‘cold‐call invitation’ to potential participants (Higginbottom et al., 2013). This approach ensured that nurse managers and RGNs with varying age, educational qualification and years of professional practice were identified and recruited to help achieve the ultimate objective of obtaining diverse and information‐rich data as desired in purposive sampling. As recommended for qualitative research, the sample size for the study was based on reaching data saturation. Code saturation, the point at which no new codes emerged, was reached after the tenth interview (Vasileiou et al., 2018). Three additional nurse managers were interviewed to confirm the findings and to seek for additional explanation to the findings where possible. This brought the total number of participants to 13. Each participant was given a simple pen as a souvenir after the interview.

### 4.6 Data collection

Data for this study were collected through in‐depth interviews aided by a semi‐structured interview guide developed through a review of the literature on barriers to NHA and nurses' identification of deterioration among hospital in‐patients NHA (Table 1). The interview guide was reviewed by the third author who has the most research experience on the team to ensure face validity. A pretest of the interview guide was done at an analogous institution. Following the pretest, slight modifications were made to finalize the interview guide before its application. Semi‐structured interviewing as the only data collection approach is appropriate for a focused ethnographic design (Cruz & Higginbottom, 2013). Data collection focused on key events during the course of nursing work including patient admission, and identification and management of deteriorating patients, as these events are the moments that are most likely to portray the true state of NHA practices. Interviews were conducted face‐to‐face within a private room in the ward setting and lasted between 20 and 70 min. All interviews were audio‐recorded and transferred to a password‐protected computer.

**TABLE 1:** Semi‐structured interview guide.

1. What do you as a registered general nurse during your shift?
2. Tell me about what you usually do when you are admitting a new patient [Probes: What patient assessment data do you usually collect? Why do you collect these types of patient assessment data? Why don't you collect other types of patient assessment data (if applicable)?]
3. What will you say about the data collected (or not collected) and your role as a nurse?
4. Tell me about one situation you think the physical condition of a patient in your ward was deteriorating (Probes: What made you think the patient's physical condition deteriorated? What did you do? Why did you do what you did? What are some of the challenges you have when communicating with doctors about deterioration in your patients?)
5. How does the ward environment enable or inhibit your nursing health assessment practice as a registered general nurse? (Probes: How does your relationship or interaction with other people or group of people in the ward influence your health assessment practice? How does the arrangement or schedule of work influence your health assessment practice? How does the material resources in the ward influence your health assessment practice?)
6. What priority do you think is given to nursing health assessment in patient care activities in this ward? (Probe: do you think nursing health assessment is given the needed priority in patient care activities in this ward?)
7. What do you think helped in shaping your current nursing health assessment practice? (Probe: Training or education in college or the university? Lack of skills? Lack of confidence? Lack of impact on patient outcomes?)
8. What other thing(s) will you like to add about nursing health assessment in your ward or hospital setting?

### 4.7 Data analysis

Data were analysed inductively using interpretive qualitative content analysis method with the help of ATLAS.ti software to reveal the latent content of the data in the form of themes (Graneheim & Lundman, 2004; Lindgren et al., 2020). The latent content involves a high degree of abstraction and interpretation to reveal the relationship aspect of the text (Graneheim & Lundman, 2004). The central focus of analysis was on the aim and context of the research with an emphasis on the differences and similarities within codes and categories.

Audio‐recording of the interviews was transcribed verbatim and constituted the unit of analysis. The audio‐recording and transcripts were listened to and read multiple times respectively, to gain a sense of the whole. The text was sorted into the state of NHA practices and explanations for the state of NHA evident in the study setting. Guided by the aim, context and theoretical framework of the study, meaning units were then identified and condensed before being abstracted and labelled as codes. The codes were compared based on differences and similarities and sorted into subcategories and/or categories (Graneheim & Lundman, 2004; Lindgren et al., 2020). The first three interview transcripts were independently coded by the first and second authors, and a coding frame was generated through discussion between the two researchers. The third author reviewed the coding frame by comparing the quotations with codes to ensure accuracy of interpretation. The coding frame was then applied to the rest of the transcripts by the first author. New codes, subthemes and themes were formulated by the first author and thoroughly discussed by the research team to achieve consensus.

### 4.8 Ethics Statement

Ethics approval was obtained from the Committee on Human Research, Publication and Ethics (CHRPE/AP/319/22) of the Kwame Nkrumah University of Science and Technology. Prior to interviews, it was ensured that participants understood the aim and procedures of the study through a face‐to‐face pre‐discussion session, and voluntarily agreed to participate in the study. Participants were free to withdraw from the study at any stage without suffering any consequences. The research team also assured the participants of anonymity, privacy and confidentiality by assigning codes to the participants. A written informed consent was obtained from all participants before interviews commenced.

### 4.9 Rigour

Triangulation of the data analysis process was adopted to enhance credibility. The first three interview transcripts were independently coded by the first and second authors, and the emerging codes discussed to arrive at the coding frame. This was then further discussed with the third author to ensure agreement and credibility of interpretation. The coding frame was then applied to the remaining transcripts by the first author. New codes that emerged were noted and discussed with the second and third authors to reach consensus. To validate the interpretation of data, the initial findings of the interviews were presented to the nurse managers of the wards included in the study and the director of nursing of the hospital to confirm the findings or to provide additional explanations where necessary. Although the first and second authors are currently practicing as nurse educators, they have experience practicing as RGNs in other hospitals within the broader study setting. This prior experience of NHA within the study setting served as an advantage in relation to data interpretation and increased trustworthiness. All participants were recruited by the second author and were then interviewed by the first author to help enhance credibility, dependability and reflexivity. Both authors have prior experience in participant recruitment and qualitative interviewing.

## 5 FINDINGS

### 5.1 Participants

A total of 13 registered general nurses were interviewed in three different wards of the hospital: general surgical ward (*n* = 4), general medical ward one (*n* = 5) and general medical ward three (*n* = 3), and the director of nursing services (*n* = 1). Table 2 presents the demographic characteristics of those who participated in the study.

**TABLE 2:** Demographic characteristics.

Participant	Gender	Age (years)	Academic qualification	Professional qualification	Working experience (years)	Rank
GMWRGN1	M	30	MPhil	RGN	4	Senior Nursing Officer
GMWRGN2	M	32	Diploma	RGN	5	Senior Staff Nurse
GMWRGN3	F	28	BSc	RGN	2	Nursing Officer
GMWRGN4	M	27	Diploma	RGN	3	Staff Nurse
GMWRGN5	F	31	Diploma	RGN	3	Staff Nurse
GMWRGN6	F	30	BSc	RGN	3	Staff Nurse
GSWRGN1	M	44	BSc	RGN	9	Principal Nursing Officer
GSWRGN2	M	34	BSc	RGN	3	Nursing Officer
GSWRGN3	M	29	Diploma	RGN	2	Staff Nurse
NM1GMW	F	46	MSc	RGN	23	Deputy Director of Nursing Services
NM2GMW	M	38	MPhil	RGN	16	Deputy Director of Nursing Services
NM3GSW	M	39	MSc	RGN	13	Principal Nursing Officer
NM4DNS	F	59	MSc	RGN	40	Director of Nursing Services

**FIGURE 1:**
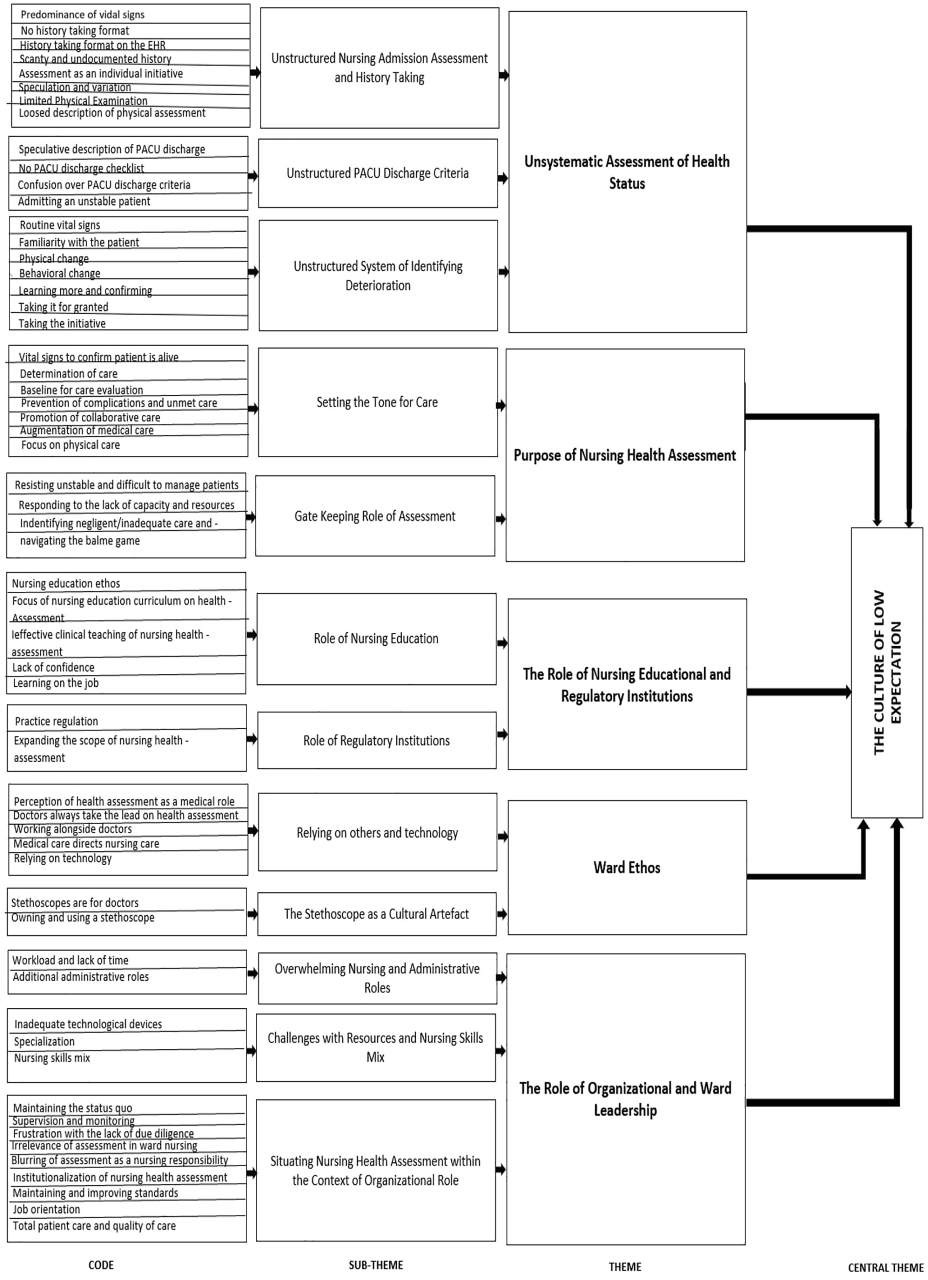
Thematic map.

A total of 57 codes were generated and aggregated into 12 subthemes. The subthemes were abstracted into five themes and a central theme. The five themes include Unsystematic Assessment of Health Status, Purpose of Nursing Health Assessment, Role of Nursing Educational and Regulatory Institutions, Ward Ethos and Role of Organizational and Ward Leadership. Details of the thematic map are provided in Figure 1 below.

The culture of low expectation was recognized as a central theme that defined and characterized RGNs' health assessment practices. The RGNs of this study were accustomed to a practice of NHA that did not seek to fully maximize the potential inherent in a NHA. The culture of low expectation within the study setting seemed to be anchored on the assumption or belief that NHA is not relevant for nursing work or practice. Although the impact of this assumption and the culture of low expectation were more visible in the wards, the wider culture of nursing education and nursing practice regulation, together with hospital organizational and ward leadership, created an atmosphere that fostered the NHA practices described in this study. Consequently, the data generated from NHA practices observed in this setting lacked a clear and vivid purpose in shaping nursing practice or work. This quote from a participant in the medical ward brings to focus the role of nursing education, practice regulation and hospital organizational and ward leadership in promoting the culture of low expectation:

I think when we get back to the ward like from school, I feel like it's the ward, what was learnt in school (about nursing health assessment) is gone but what we've come to meet, we have to continue it like that. (GMWRGN1)

### 5.2 Theme 1: Unsystematic assessment of health status

There was no clear standard established by the wards, hospital or regulatory body on how nurses should assess patients. The approaches used by nurses to assess patients during ward admission, post‐anaesthetic care unit (PACU) discharge and on recognition of deterioration varied from one nurse to the other.

#### 5.2.1 Unstructured nursing admission assessment and history taking

Vital signs were the most certain, and in some instances, the only health assessment data a nurse would obtain when admitting a new patient into the ward. The interpretation or meaning of the vital signs recorded did not usually provoke the need for further data collection either through history taking or physical assessment.

…in this ward, our assessment is just always limited to the vital signs, after the vital signs that's all, if the parameters are good or not good. (GMWRGN1)

Descriptions of other health assessment data obtained from patients during admission were speculative and varied with the use of the terms ‘sometimes’, ‘maybe’ and ‘at times’. A wide range of other health assessment data were obtained using observation and direct questioning. Although some nurses stated that they did not take health history, some health history could be obtained in a non‐standardized manner usually occurring throughout the entire period of nurse–patient relationship and care provision, and not necessarily at the time of admission.

…you can take history let's say with one‐on‐one conversation with the patient. When you are having your normal conversation or something like that, you can ask some few questions. But mostly the history‐taking which needs documentation is being done by the doctors. (GMW3RGN2)

This discretionary approach to history taking often yielded scanty, undocumented data. A nurse manager also hinted that the hospital record keeping system had no provision for RGNs to document their assessment finding:

Apart from the nurses' notes where you will just do some one‐two, and write on what you have… but it doesn't provide any sheet where you can do your comprehensive assessment and really spell it out. (NM2GMW)

On the use of physical assessment skills, these skills were not routinely used by nurses, either on admission or as part of ongoing patient assessment and care. Physical assessment was often used loosely with reference made mostly to the technique of inspection. A more explicit use of the skills of physical assessment was often associated with checking correct placement of a nasogastric tube during the process of tube feeding. According to the nurses, this was not even done by all nurses.

…yesterday we had a case of patient's NG tube not being in‐situ and someone might try to feed. We had a stethoscope which belonged to a nurse, so we checked with the stethoscope. Many nurses might not even check these things. Someone might just come oh medications must be served via the NG tube then they start serving. (GMWRGN3)

#### 5.2.2 Unstructured PACU discharge criterion

Post‐operative patients were picked up and transported from the PACU (also referred to as recovery) to the general surgical ward by RGNs when informed by the PACU staff that a patient was stable enough to move to the ward. In some instances, the RGNs disagreed with the assessment of the PACU staff and hence may return to the ward without the patient. This was because there was no clearly documented PACU discharge criteria put in place by the hospital or the wards. Although the nurses were mostly speculative about what assessment was done prior to receiving a patient from PACU, they insisted that the ward had taken time to train all nurses on how to assess and receive a patient from the PACU.

Checklist before taking up? It is not on a sheet but the ward has a protocol which is not on a sheet but we know that when we get there, we have part of our training that we have been given, that when we get there, we have to make sure that the vitals are ok, the patient has fully recovered… (GSWRGN3)

The absence of a PACU discharge criteria sometimes created confusion both within the ward staff, and between the staff of the ward and PACU when deciding on the general stability of patients to be transferred to the ward. This also meant that some patients may be transferred to the ward although the ward staff may perceive them as unstable or unfit to be transferred from the PACU. Nurses who transported patients deemed as unfit to be transferred to the ward from the PACU were chastised by their colleagues.

#### 5.2.3 Unstructured system of identifying deterioration

The hospital and the wards did not have a system in place to promote the early recognition and response to deterioration among in‐patients. Nurses identified deterioration using routine observation of vital signs and, with some level of discretion, observation of other physical and behavioural changes. An important factor that helped nurses to recognize changes in physical or behavioural patterns was the nurse's familiarity with the patient.

Per my closeness to the patient am able to tell that the patient is not the way I took over and that will prompt me to check those respective vital signs and then confirm. (GSWRGN2)

The idea of identifying deterioration seemed to be taken lightly and for granted as if the skill required to identify deterioration came easily to all RGNs when observing physiological changes. Vital signs and other parameters were also checked to confirm and to learn more about a change in physical or behavioural patterns. The nurses responded to deterioration using independent actions such as initiation of oxygen and intravenous fluid therapies if medical doctors were not readily accessible. These practices were conventions, and not necessarily documented protocols or guidelines, but were nonetheless endorsed by the hospital leadership as long as they did not compromise patient safety.

### 5.3 Theme 2: Purpose of nursing health assessment data

The findings of nurses' health assessment practices were used to support patient care activities. The relationship, however, between NHA findings and initiation of nursing care activities was not very clear. There was also a general sense of an underwhelming utilization of NHA in nursing care activities. The idea of holistic care, including all aspects of human functioning and need, was not apparent in the practice of the RGNs. It would seem that nurses focused on the provision of physical care, which required little to no independent nursing assessment of health status, or using NHA to augment medical care.

#### 5.3.1 Setting the tone for care

The nurses showed awareness of the potential of NHA for nursing care and patient outcomes. The findings of NHA provided the baseline for care, determined nursing interventions and enabled care evaluation.

As a nurse, I feel like without the nursing assessment tool then your practice is to be questioned because that is one of the major things that should make you actually a qualified nurse because every activity of the nurse goes back to assessment. (GMWRGN4)

On the contrary, nursing practice in the setting did not overtly involve the formulation of nursing diagnoses as envisioned in the nursing process and the holistic model of care.

When you come to the hospital, a new patient comes in you take the data of the patient and you build a (nursing) care plan for the patient and from there you evaluate and all that but err because we don't do it here so it is just like you come to work you are serving medications and all that. (GMWRGN3)

Nurses described the purpose of the assessment data in general terms without clearly demonstrating how that data can be used to initiate or direct nursing care activities. Nursing interventions were initiated without a plan of care firmly supported by NHA data documented in the patient's health records. Overall, health assessment data could be collected, analysed and used to initiate an intervention without any documentary evidence of the assessment finding, a nursing diagnosis or even the nursing intervention itself.

If I am admitting a case, then I check vital signs, probably I know a patient is saturating at 92% or 90%. Then I as a nurse I know a patient saturating at that level is not a good value for a normal patient. So, I as a nurse I know that this person needs oxygen. I need to prop up this patient in bed and probably I need to monitor the vitals regularly. (GMWRGN1)

This form of utilizing health assessment findings could find expression in both collaborative and independent nursing roles. As an example, a nurse explained how a newly admitted patient could be helped to sleep through an independently initiated nursing intervention without an explicitly documented health problem or nursing diagnosis.

…maybe you tell me you have difficulty in sleeping at night. Then I will now ask you further questions to find out why you have difficulty in sleeping then I can help you with something maybe it is the environment you are not used to. It is not every time you need medications. (GMWRGN3)

There were also very direct descriptions of NHA being used to augment or even sometimes to initiate a medical intervention.

… sometimes per the observation, if somebody needs blood per our observation, nursing observation of the conjunctiva and those things, then we prompt them to do full blood count or at least HB to know the state of patient's blood. Then if the HB is low then they transfuse accordingly. (GSWRGN2)

The nurses also posited that a more purposeful and thorough NHA could help improve interdisciplinary collaboration and improve patient outcomes. Some nurses stated how the current NHA practices limited interdisciplinary collaboration and could heighten the possibility of not meeting all the care needs of the patient. This reality was expressed by a nurse when discussing how a routine 4‐hourly feeding pattern could be individualized to meet the specific needs of a patient on nasogastric tube feeding based on the nurse's health assessment of the patient and subsequent collaboration with other healthcare professionals:

We have a four hourly feeding pattern but this average interval is not working for all the patients. So, at some point they might need the dietician to come in. So, it's like a patient will come with obvious need but the nurse being kind of that coordinator wouldn't show until a later date when probably a doctor comes and then during discussion, the need to even invite a dietician, the feeding plan for the patient will come up. The nurse at the onset could have brough this up, so by the time the patient is put on it probably some amount of harm has already been caused. (GMWRGN2)

The lack of a thorough assessment of other domains of health and functioning such as the socioeconomic status, cultural background and religious affiliation was also identified as contributing to the predominant focus on physical care rather than holistic care.

It is like the background checks of the patient, sometimes we do forget about the kind of lifestyle the patient was living before he came for admission and I'm not sure it's part of the assessment we do here. We just concentrate that the patient has presented with the condition and we are treating the condition. (GSWRGN3)

Some of the RGNs seemed to hold in high esteem the provision of physical care in comparison with a thorough health assessment and holistic care provision. The organizational and ward culture seemed to have endorsed and limited the focus of the nurses to the provision of physical care. In the excerpt below, the nurse captures the role expectation and the focus on physical care quite vividly in relation to the interpretation of an electrocardiogram:

I was asked by let me say an organization to interpret ECG, electrocardiogram and I told them that here we don't do it because we our task says just send the patient there, do the procedure, we bring the patient back. Nurses are not supposed to interpret. …so that's the system that we met and we are fixing ourselves in the system. (GSWRGN3)

#### 5.3.2 The gatekeeping role of nursing health assessment

In the surgical wards, the RGNs narrated how they resisted the admission or transfer‐in of patients who were perceived as unstable or difficult to manage coming either from the Accident and Emergency Unit, or PACU.

…they were trying to push her to the ward and together with Akpaku (not actual name), we resisted that. No! somebody has cardiac arrest twice on operation bed, you don't bring the person to ward, ICU is the best place to send the patient. (GSWRGN1)

This resistance to the admission of perceived unstable patients was a direct response to the lack of resources or capacity to manage such patients in the ward when complications arose.

This was a patient typically you could tell that they needed intubation, we don't have it here. (GMWRGN2)

The nurses also used their health assessment findings to identify poor care and to navigate the blame game that seemed to point the finger at nurses whenever a patient's condition deteriorated or an adverse patient care outcome was recorded. The health assessment of nurses was thus deliberate to pick up poor or negligent care by other health professionals or from nursing colleagues in a different ward. The general surgical ward had this practice of tracking post‐operative wounds to determine the origin of surgical site infections to exonerate nursing staff:

…we have a system here, tracking post‐surgical infections. So, we will know whether the state in which the patient came from the theatre, there was already a sign that the due diligence wasn't done or out of nursing lapses the fault is from the ward. (GSWRGN2)

In the general medical ward, the focus was on identifying poor or negligent care from colleague nurses in other wards of the hospital, and to reduce or share the burden of care accruing from the negligence or poor care.

If you don't assess, you wouldn't even know the patient has catheter, yes am trying to be very frank here, the handing over and taking over is not very effective, so if you don't make sure you get to know the necessary things about the patient, the person will hand over and go then you see these things. (GMWRGN2)

### 5.4 Theme 3: The role of nursing educational and regulatory institutions

The quality of nursing education and standards of nursing practice regulation in the study setting did not appear to place priority on NHA as a critical skillset for a registered general nurse. The manifested quality of nursing education and standards of practice regulation (cultural artefact) inversely influenced the belief of NHA as an integral part of nursing (assumption and values), and instead, tacitly entrenched the assumption that NHA is not relevant for nursing work or practice.

#### 5.4.1 Role of nursing education

Nursing educational experience was not designed to project NHA as a significant educational outcome. It seemed that nursing students, and registered general nurses by extension, were not socialized to inculcate NHA as a mandatory and routine part of nursing practice.

…throughout the training here, they make it look like those things are not to be done by nurses, so before the person comes out of the training and starts working, they don't see the need, the importance of you may be doing auscultation, palpation and those things. If you ask our students on clinicals right now, I'm sure you won't get anybody with stethoscope. (GSWRGN1)

The curriculum utilized in nursing education institutions was described as having a narrow focus on NHA. In some instances, students were only taught about the different techniques of physical assessment without a corresponding demonstration of how the different techniques could be used for an in‐depth assessment of different regions or systems of the body. Where nursing educators attempted to teach regional or system assessment, the theoretical component of NHA was usually taught without adequate preparation and opportunities for students to develop proficiency in skills laboratories or clinical settings.

Nursing assessment was taught but I think it was just shallow. We did the theoretical part of it but the practical part of it wasn't done in the skills lab. (GMWRGN3)

Because of the limited opportunities to practice and develop proficiency in NHA skills, registered general nurses did not have the confidence to use these skills in their everyday practice.

…because practically I haven't actually practiced that to be very sure that I can do it, I wouldn't want to play the try and error thing, yes, but those that you are very good in, you would actually want to do it. (GMWRGN4)

To adapt to the demands and expectations of colleagues within the ward, some nurses had to learn NHA skills on the job. Nurse managers in some wards took time to properly orient newly graduated nurses on some of the job expectations and thus facilitated learning.

I learnt how to use a stethoscope to check if an NG tube is in situ when I started working in the ward, I should have known that before coming to the ward. (GMWRGN3)

#### 5.4.2 Role of regulatory institutions

There was some sense of understanding that the scope and standard of nursing practice promulgated by the regulatory body of nursing in the study setting did not include thorough NHA. In highlighting the role of the body responsible for regulating nursing practice in changing the prevailing NHA practice, the need to expand the scope of nursing to include an extensive NHA was recommended:

…since the system is changing, its high time that they (regulatory body) have to update the kind of (assessment) procedures that nurses are supposed to do. (GSWRGN3)

An important emphasis was placed on the need for this expansion to include a routine assessment of the socioeconomic domain of health and functioning.

…probably something that we don't usually give much attention to is whether the patient has a support system. I think it's very important but it's something that we don't usually give a lot of attention… (GMWRGN2)

The ease with which newly RNs passed through the educational system and the licensing examination, without the requisite NHA competencies, was also a demonstration that NHA was not a priority in terms of the scope of practice for registered general nurses.

Yes, they have gone through, obviously they have gone through (nursing school and licensing) successfully but if you ask them, how do you assess a patient, they don't know. If you mention a term related to lung assessment they don't know, they've not heard before. (GMWRGN2)

### 5.5 Theme 4: Ward ethos

The perception of health assessment as a predominant medical role limited nurses' history taking and use of physical assessment skills in the wards. The manifestations of some of these perceptions had implications on even the ownership and use of a stethoscope among nurses.

#### 5.5.1 Relying on other professionals and technology

The conduct of a systematic health assessment comprising of a structured history and a focused physical assessment was perceived as a preserve of the medical profession. Some registered general nurses believed that using physical examination skills was outside the scope of practice of a registered general nurse. This perception enabled registered general nurses to rationalize the prevailing NHA practices observed in the study setting.

Honestly, you know in this country, yeah, most of the things are perceived to be for doctors so honestly speaking some of the things are not being done here. (GSWRGN1)

I think maybe it's not part of our scope of practice here in a teaching hospital. I've been here, I've never seen a nurse checking for the bowel sound, I've never seen such things. (GSWRGN3)

Some of the participants stated that because patients arrive in the wards after consultation with the medical doctor, it would be a needless duplication of the health assessment since the medical doctors always take the lead to obtain a health history and perform a physical assessment.

…sometimes to me, I feel like the necessary assessment has been done before the patient gets into the ward. (GMWRGN1)

The constant presence of medical doctors in the wards, working alongside nurses, also meant the nurses could conveniently leave out the health assessment of patients to the medical doctors.

As a consequence of these perceptions, registered general nurses relied extensively on the health history and physical assessment data obtained and documented by the medical doctors to guide their nursing interventions. The extent to which the nurses relied on the health assessment of medical doctors is highlighted in a response to a probing question:

MAS So in the absence of the doctor's assessment, what do you think would happen?

GMWRGN3 Care cannot be provided because the doctor will have to assess, find out a diagnosis which we will work on. So, if the doctor doesn't do it then what we would basically do as a nurse, is limited.

An alternative to obtaining patient health data was to rely on technology. This came in the form of patient monitoring machines and made it easier to obtain patient's vital signs. This was especially utilized when monitoring a high acuity patient.

#### 5.5.2 The stethoscope as a cultural artefact

The use of the stethoscope as a device was also perceived as largely exclusive to medical doctors. As a result, most of the nurses at the study site did not own personal stethoscopes.

Honestly… when you get to the ward right now, you won't see any nurse with his or her personal stethoscope. (GSWRGN1)

It was hinted that the nurses did not own and use stethoscopes because they did not have the level of proficiency needed to confidently wield and use it as a tool of work.

So, even in schools if we were enlightened more on the use of stethoscope on a patient it would have helped because is like we have that general mindset that stethoscopes are for doctors so we hardly use them. (GMWRGN1)

A legitimate use for the stethoscope among nurses in the study setting was to listen to Korotkoff sounds while checking blood pressure with an aneroid or mercury sphygmomanometer. As at the time of data collection, the hospital had phased out mercury and aneroid sphygmomanometers in place of electronic or digital blood pressure apparatus.

…we perceive those things, the use of stethoscope to be for the doctors because we don't use the mercury type (sphygmomanometer) we just think it is about checking the BP that's when you need a stethoscope… (GMWRGN3)

Nurses who used the stethoscopes for purposes other than listening to Korotkoff sounds felt uneasy about it. A nurse who was trained outside of the study setting described his/her use of the stethoscope as odd among other nurses.

…I have my stethoscope but even when somebody sees you with stethoscope, when I started working, …but I realized it seems like I'm the odd person. (GSWRGN1)

### 5.6 Theme 5: The role of organizational and ward leadership

The planning and delivery of nursing care within the hospital as an organization, and the ward environment implicitly promoted the NHA practices observed in the study setting. The conduct of managers and leaders at all levels of the hospital organization seemed indifferent to the quality of nurses' health assessment practices.

#### 5.6.1 Overwhelming nursing and administrative roles

There was a short period of time available to registered general nurses within a shift to provide nursing care for a large number of patients and to fulfil additional administrative responsibilities. Nursing care was provided within 2 day shifts of 6 h each and a night shift of 12 h. This circumstance compelled the nurses to prioritize physical nursing care and administrative roles over NHA.

Looking at the number of patients we have in the ward, so probably I have about seven or eight patients… critical cases to attend to. Then a new case comes, I just have to do the basic ones (assessment) and continue with my other nursing care, I don't really have that time to do the proper head‐to‐toe assessment. (GMWRGN1)

#### 5.6.2 Challenges with resources and nursing skills mix

The non‐availability of resources needed to facilitate assessment of health status was another factor identified as limiting nurses' health assessment practices. This challenge occurred within a wider system of resource limitation peculiar to the setting. The examples commonly stated included the limited availability of technological devices and or their accessories such as monitors, stethoscopes and glucometer strips.

The number and professional qualification of the nurses in the ward also contributed to the state of NHA observed in the wards. A good number of nurses posted by the central government to the hospital, and further assigned to the wards by the hospital organizational leadership, were nursing health assistants and mental health nurses. This cadre of nurses do not have the knowledge and skills required to assess, formulate nursing diagnoses and develop a nursing care plan for patients with medical‐surgical health problems.

…I got to realized that it was not only about capacity but the problem is the nature of cadres that come in. Naturally those who are from the RGN background do not have so much problem with that but you see people from the RM (registered mental health nurse) and EN (enrolled nurse), they are a big problem. (NM2GMW)

#### 5.6.3 Situating nursing health assessment within the context of organizational role

The state of NHA practices observed in the study setting was considered as the norm. These practices had become so entrenched that some participants could not clearly explain why the observed NHA practices were considered as the norm beyond admitting that they were simply maintaining the status quo.

…so, you've come to meet it (assessment practices) but low key at times you feel like, no this is not the right thing because you know what you are supposed to do. You just have to go extra but you've come to meet it like that rotating in the ward so just play along. (GMWRGN3)

The notion that workloads and lack of resources sometimes prevented nurses from thoroughly assessing a patient and documenting same was dismissed as untenable. It was noted that assessment practices described in this study were still enacted even when nurses had significantly reduced workload.

…because we have done it for a period of time, it is like it has become part of us even if you are free and they bring the patient you still do what you've been doing. (GMW3RGN1)

The culture of supervision and monitoring in the wards did not appear to give top most priority to NHA practices and other clinical nursing roles but rather to non‐clinical expectations such as regular job attendance and adherence to the prescribed uniforms.

You will not see someone coming to supervise what we are doing. There is no one supervising anyone on some of these things. They only come around just to check uniforms and to see those who are on duty. (GMWRGN1)

Some attempts were made by nurse managers to have nurses utilize, not necessarily NHA but, the nursing care plan that was incorporated into the electronic health records of the hospital. This demand was made of the nurses without a corresponding demand to assess the health status of patients and to show evidence of an assessment of health status through documentation of the findings of the assessment in the patient's health records. Through this lapse, the attempts to implement the nursing care plan became an exercise to satisfy nurse managers rather than to systematically address patients' needs. Aside formulating incorrect nursing diagnoses, which the nurse managers found embarrassing, some nurses planned care for patients that did not reflect the health problems and care needs of the patients.

What I have detected with the nursing care plan is that they do it to satisfy the ward. Sometimes they are not true reflections of the patient's problems. (NM1GMW)

Others simply copied their previous care plans for the same or a different patient without really ascertaining the current health problems and care needs of the present patient. In the case of nursing health assistants who were also assigned to patients and expected to plan care for these patients, they had no option but to rely on their colleagues with the registered general nursing background since their educational preparation did not involve the planning of care for patients. All these observations put together demotivated some nurse managers, and efforts put into supervision and monitoring gradually reduced.

I was on the nurses to start writing it (nursing diagnoses), then one nurse came out with a funny diagnosis that I was taken aback actually… it was so shameful. (NM2GMW)

Within this practice environment, nurses who were vigilant and diligent in their assessment and discharge of nursing care responsibilities expressed their frustration with the general atmosphere that accepted and promoted the status quo. Such nurses were also labelled as difficult to work with by some of their colleagues.

So that's why some of us, they see us to be difficult because I will make sure that I ask the necessary questions in your presence. And I remember there are instances somebody will bring a patient, the patient is soiled, they just want to hand over and go. I will assess… (GMWRGN2)

These happenings, together with the unstructured approach to NHA, suggested that NHA had not been adequately institutionalized as a significant function of the nursing role. The hospital organizational and ward leadership were identified as responsible for institutionalizing NHA as a legitimate and necessary nursing role, and hence, the prevailing NHA practices observed in the wards.

MAS The state of Nursing Health Assessment in this ward, in your opinion, what do you think is the most important factor that has shaped it in this way?

GMWRGN2 I will say a kind of lukewarm attitude of the leadership. They are not stern enough to establish the rules and insist adherence to this.

One nurse concluded that NHA was not valued and appreciated as much as it should be.

I think some people don't really see the importance of assessment… I know some others don't see the importance of doing the head‐to‐toe or the proper physical assessment. (GMWRGN1)

It was also noted that standards of nursing care practices were maintained, if not improved, when the ward leadership purposefully and intentionally stepped up their supervisory and monitoring roles. Such actions ensured that nurses discharged their care responsibilities fully without neglecting obvious physical care needs.

…I think because we don't do the head‐to‐toe examination, we will see patient with long nails, we don't care. They taught us in school… we don't do because we don't even add to our priorities like this is what I want to make patient look. So, at a point our in‐charge insisted that night staff have to help patients bath every morning and that was what was happening… but if she gets reluctant on those things you can see a patient very dirty, they will all be there everybody will come serve medication hmmm. That's the reality on the ground. (GMW3RGN1)

## 6 DISCUSSION

The aim of this study was to describe RGNs' health assessment practices, and to explore the assumptions and values that influenced the assessment practices within the setting. Our findings showed that RGNs' health assessment practices were generally unsystematic, unstructured and with varied, different assessment data collected alongside vital signs. There was no nursing history‐taking framework and physical assessment skills were not routinely used by the RGNs to collect data on the health status of patients. Our findings also suggested that the purpose of the RGN's health assessment was not clearly delineated and widely acknowledged by all stakeholders of nursing practice. This lack of a clear purpose implied that RGNs could go about nursing work without a need for a thorough assessment of the patient's health status. We argue that central to this state of NHA is the culture of low expectation on NHA. Important stakeholders of nursing practice such as regulatory institutions, nursing education and practice settings have become accustomed to the prevailing state of NHA. Despite awareness of the value of NHA to nursing practice, the RGNs within this study setting experienced a gradual reshaping of their assumptions or values about NHA to align with the predominant social or material reality.

Several authors have reported on the limited use of physical assessment skills and associated NHA data obtained by RGNs across the globe over the last four decades (Morrell et al., 2021; Osborne et al., 2015). Research on nurses' use of physical assessment skills and barriers associated with the use of physical assessment skills in clinical settings has generated much interest among nursing scholars (Maniago et al., 2021) with little focus given to how these skills are integrated into everyday nursing practice. The lack of a clear purpose for physical assessment in nursing was identified as a significant barrier against nurses' use of physical assessment by Tan et al. (2021) in their systematic review of the literature.

A holistic NHA, including physical assessment, is considered an important entry‐level competence for registered general nurses (Nursing and Midwifery Council of Ghana, 2022). This study highlighted a culture of nursing education and practice that did not emphasize NHA as an important competence required for everyday clinical nursing practice. The role of nursing education in terms of curriculum adequacy, pedagogy and clinical learning experiences has been identified as a contribution to the unsatisfactory state of NHA observed in clinical settings (Maniago et al., 2021; Tan et al., 2021). In Douglas et al. (2015), about 70% of the skills were never taught or performed by students, and students perceived physical assessment skills as marginalized not only in clinical settings but also in the university. Participants in this study attributed the rare use of physical assessment to low confidence arising out of inadequate educational preparation. With most nursing programs teaching NHA under the biomedical model and emphasizing comprehensive physical assessment and medical diagnosis (Lesa & Dixon, 2007; Zambas, 2010), RGNs are bound to be constrained as to what to make of their assessment findings since the formulation of a medical diagnosis is not a foremost responsibility of the nurse. The role of nursing regulatory institutions has not been scrutinized thoroughly in the literature, and as the institutions mandated to set standards for nursing education and practice, the current state of NHA seems to suggest that regulatory institutions may also have a low expectation of NHA and what nurses do with their assessment findings. While we acknowledge that the assessment and evaluation of NHA competences as part of nurse licensing may be difficult, situating competence assessment within a clearly defined framework of patient health assessment with a clear purpose may improve the ease of competence assessment.

The perception that physical assessment is the exclusive job responsibility of the doctor has also been cited in previous studies (Tan et al., 2021). Underneath this perception is the assumption that a structured health history and physical assessment is not relevant for nursing work. Other findings reported in the literature such as high workloads and lack of time or interruptions (Tan et al., 2021) further support this assumption. If nurses are pre‐occupied with high workload and do not have time to do a health assessment, it then presupposes that nursing work can be done without a structured nursing health assessment. Nursing work driven by an unstructured approach to health assessment can only focus on traditional and routine nursing care activities. This explains why the RGNs in our study focused on the provision of routine physical care, that required little to no health assessment, rather than holistic patient care. An unstructured approach to health assessment is not only invisible but also raises concerns about the depth and breadth of health assessment, health problems identification, quality of nursing care and patient safety. Moreover, if RGNs are relying on the assessment data of other healthcare professionals, nursing scholars need to be interested in how these assessment data are incorporated into nursing care and its impact on quality of care and patient safety.

It is also widely documented that physical assessment skills performed by nurses were usually confined to skills mandated by organizational or ward culture (Morrell et al., 2021; Tan et al., 2021). Based on the state of the evidence on nurses' use of physical assessment skills in clinical settings, it could be argued that hospital organizational and ward leadership may be accommodating, promoting and normalizing this practice through its continuous indifference. In other studies, working in a specialized unit had a positive influence on nurses' use of physical assessment skills (Cicolini et al., 2015; Fennessey, 2016). It is possible that the influence attributed to the speciality units could be a product of the culture of these units aside the obvious care requirements imposed by the tag of a specialty unit. The state of NHA in our study setting has been attributed to the indifference of organizational and ward leadership towards NHA in terms of prioritization, professional development and monitoring and supervision.

Notwithstanding the fact that all stakeholders of nursing unanimously agree on the potential benefit of NHA to nursing practice and patient outcomes, there appears to be a lack of consensus on the role and purpose of NHA within nursing practice. The culture of low expectation found in this study is anchored on the lack of a clear purpose and the seeming irrelevance of NHA data on nursing care activities. At the core of nursing care is the diagnosis and treatment of human responses to potential or actual problems (Carpenito, 2021). Nurses therefore should not shy away from enacting nursing practice being guided by nursing diagnoses. Based on findings in our study settings and elsewhere, this does not seem to be the case. In their review of health records in burns wards to describe and ascertain the formulation of nursing diagnoses reflective of the care needs of patients, Khajehgoodari et al. (2020) found that patient care needs were not assessed and documented by nurses, and nursing diagnoses formulated without regard to the actual or potential health problems of patients. They therefore concluded that there was no nursing thinking behind the observed nursing work given that nurses provided nursing care as requested by physicians. To fully realize the potential of NHA, several authors have called for the incorporation of NHA within a holistic assessment framework (Lesa & Dixon, 2007; Tan et al., 2021). It would seem however that the early recognition of deterioration among in‐patients rather than the formulation of a nursing diagnosis may also be considered as an outcome of NHA (Douglas et al., 2016). We argue that nurses can confidently own their assessment and use it to provide patient‐centred care, including the early recognition of deterioration, when NHA is incorporated into a holistic assessment model such as the functional health patterns (Carpenito, 2021). Our study also provides supported for the lack of a nursing health history framework, which quite conspicuously has not received the same attention as physical assessment. As the most important constituent of the diagnostic process, nursing needs to adopt and standardize a common framework to ensure uniformity and efficiency in its use.

### 6.1 Limitations

The participants for this study were recruited from a single hospital and the culture of this hospital may not be the same as other hospitals within the broader study context. Although the registered general nursing curriculum does require registered general nurses' to be taught NHA, there is no clear evidence of what and how NHA is taught in the wider study setting. Moreover, the study did not include views from nursing education and regulatory institutions.

### 6.2 Recommendations for further research

Future research should include multiple study sites and seek the perspectives of nursing education and regulatory institutions. Furthermore, this study used only semi‐structured interviewing to collect data. The findings could have been enriched if participant observation was included as a data collection approach.

## 7 CONCLUSION

Healthcare systems across the world currently are under utilizing the potential contribution of NHA to nursing practice and patient outcomes. The full potential of NHA may be realized when it is construed as a truly independent nursing activity with a clearly defined purpose. To reduce the culture of low expectation associated with NHA, there is the need for stakeholders of nursing education and practice to agree on what nurses do with their health assessment findings and to consciously institutionalize it as part of nursing practice. We join other researchers in proposing that NHA be situated within a holistic model of assessment such as the functional health patterns and nursing diagnoses formulated out of the health assessment of nurses. Our findings suggest that leadership at both the organizational and ward level can significantly influence NHA practices. We recommend that organizational and ward leadership take the initiative to adopt holistic NHA tools, encourage and reward its use within practice settings.

## AUTHOR CONTRIBUTIONS

Mohammed Awal Salifu and David Abdulai Salifu designed the study. David Abdulai Salifu recruited the participants, and Mohammed Awal Salifu conducted all interviews. Data were analysed by Mohammed Awal Salifu and David Abdulai Salifu. The final report was drafted by Mohammed Awal Salifu with both David Abdulai Salifu and Janet Gross making critical inputs and revisions to the manuscript. Janet Gross supervised and provided support during all phases of the study including data collection and analysis. All three authors read and approved the final manuscript.

## FUNDING INFORMATION

This research received no specific grant from any funding agency in the public, commercial or not‐for‐profit sectors.

## CONFLICT OF INTEREST STATEMENT

The authors declare no conflicts of interest.

## ETHICS STATEMENT

Ethics approval was obtained from the Committee on Human Research, Publication and Ethics (CHRPE/AP/313/22) of the Kwame Nkrumah University of Science and Technology. Prior to interviews, it was ensured that participants understood the aim and procedures of the study through a face‐to‐face pre‐discussion session, and voluntarily agreed to participate in the study. Participants were free to withdraw from the study at any stage without suffering any consequences. The research team also assured the participants of anonymity, privacy and confidentiality by assigning codes to the participants. A written informed consent was obtained from all participants before interviews commenced.

## Data Availability

The data that support the findings of this study are available on request from the corresponding author.
